# The impact of COVID-19 on the mental health and well-being of ambulance care professionals: A rapid review

**DOI:** 10.1371/journal.pone.0287821

**Published:** 2023-07-11

**Authors:** Remco H. A. Ebben, Tim Woensdregt, Etty Wielenga-Meijer, Thomas Pelgrim, Annet de Lange, Sivera A. A. Berben, Lilian C. M. Vloet

**Affiliations:** 1 Research Department of Emergency and Critical Care, HAN University of Applied Sciences, School of Health Studies, Nijmegen, the Netherlands; 2 Research Department Human Resource Management, HAN University of Applied Sciences, School of Organisation and Development, Nijmegen, the Netherlands; 3 Department of Psychology, Universidade da Coruna, A Coruña, Spain; 4 Faculty of Psychology, Open University Heerlen, Heerlen, Netherlands; 5 University of Stavanger, Norwegian School of Hotel Management, Stavanger, Norway; 6 Faculty of Psychology, Norwegian University of Science and Technology, Trondheim, Norway; 7 Radboud University Medical Center, Radboud Institute for Health Sciences, IQ healthcare, Nijmegen, The Netherlands; Xiamen University - Malaysia Campus: Xiamen University - Malaysia, MALAYSIA

## Abstract

The COVID-19 pandemic has a significant impact on the health and well-being of all healthcare professionals. However, for ambulance care professionals it is unknown on which health outcomes the impact of COVID-19 is measured, and what the actual impact on these health outcomes is. Therefore, the aim of this study was to gain insight in a) which type of health outcomes were measured in relation to the impact of COVID-19 among ambulance care professionals, and b) to determine the actual impact on these outcomes. A rapid review was performed in PubMed (including MEDLINE) and APA PsycInfo (EBSCO). All types of study designs on health and well-being of ambulance care professionals were included. Selection on title an abstract was performed by pairs of two reviewers. Full text selection, data extraction and quality assessment were performed by one reviewer, with a check by a second independent reviewer. The systematic searches identified 3906 unique hits, seven articles meeting selection criteria were included. Six studies quantitatively measured distress (36,0%) and PTSD (18.5%-30.9%), anxiety (14.2%-65.6%), depression (12.4%-15.3%), insomnia (60.9%), fear of infection and transmission of infection (41%-68%), and psychological burden (49.4%-92.2%). These studies used a variety of instruments, ranging from internationally validated instruments to self-developed and unvalidated questionnaires. One study qualitatively explored coping with COVID-19 by ambulance care professionals and reported that ambulance care professionals use five different strategies to cope with the impact of COVID-19. There is limited attention for the health and well-being of ambulance care professionals during the COVID-19 pandemic. Although the included number of studies and included outcomes are too limited to draw strong conclusions, our results indicate higher rates of distress, PTSD and insomnia compared to the pre-COVID-19 era. Our results urge the need to investigate the health and well-being of ambulance care professionals during and after the COVID-19 pandemic.

## Background

The COVID-19 pandemic has a significant impact on the health and well-being of all healthcare professionals. Systematic reviews and meta-analyses reported prevalence rates of 13.5%-44.7% for depression, 12.3%-41.2% for anxiety, 5.2%-56.5% for acute stress reaction, 7.4%-37.4% for post-traumatic stress disorder (PTSD), 33.8%-44.0% for sleep disorders, and 3.1%-43.0% for burnout [1–5]. Within this group of healthcare professionals, females and nurses seem to report higher risk for developing mental health outcomes compared to males and physicians (3,6). Furthermore, underlying illness, concerns about family, fear of infection, lack of personal protective equipment (PPE), working experience, lower social support, stigmatization, workload, and close contact with COVID-19 patients are risk factors for adverse mental health outcomes [4,6,7].

A specific group working within healthcare consists of professionals in the chain of emergency care: ambulance care, emergency department (ED), and intensive care units (ICU). These professionals are in the frontline of the pandemic, and were the first to be confronted with COVID-19 patients. For ICU and ED settings, prevalence rates for depression and PTSD are in range with prevalence rates of general healthcare professionals [8]. However, reported burnout prevalence rates are higher, with a negative impact on quality of life [7,9].

For the chain of emergency care, the available literature seems to be focused on the health of ICU and ED professionals, with the ambulance setting relatively underexposed. This is despite the high prevalence rates of general psychological distress (27.0%), anxiety (15.0%), depression (15.0%), PTSD (11.0%), sleeping problems (20–27%), and burn-out (8.6%) among ambulance care professional in the pre-COVID-19 era [10–12]. These high prevalence rates affect ambulance care professionals’ well-being by increasing the risk for sick leave and suicidal thoughts [10,11].

However, for ambulance care professionals, it is unknown on which health outcomes the impact of COVID-19 is measured, and what the actual impact on these health outcomes is. This insight is essential to develop and implement tailored interventions to support these professionals and to prevent the development of mental health disorders and drop-out, on top of their already increased risk. Therefore, the aim of this study was to gain insight in a) which type of health outcomes were measured in relation to the impact of COVID-19 among ambulance care professionals, and b) to determine the actual impact on these outcomes.

## Methods

### Design

A rapid review of the literature was performed according to the recommendations of the Cochrane Rapid Reviews Method Group [13]. This review is reported in concordance with the PRISMA-statement [14]. A study protocol was not registered prior to conducting the Rapid Review.

### Literature search

Firstly, the PROSPERO database and Cochrane database for systematic reviews were searched with the terms [(Ambulance OR EMS) AND Covid] for protocols and existing reviews, no records were identified. Secondly, systematic search strategies were constructed and tested with involvement of an information specialist (TP). Final searches were performed in PubMed (including MEDLINE) and APA PsycInfo (Ebsco) in October 2021. Search strategies were structured to represent ‘terms for professionals OR settings’ AND ‘terms for COVID-19’ AND ‘terms for health outcomes’. For professionals and setting we constructed broad search strategies within the chain of emergency care as studies for ambulance care might be part of larger studies. Full search strategies per database are given in S1 Appendix. Searches were restricted by year of publication (≥ November 2019) due to the first emergence of COVID-19.

### Study inclusion criteria

We included systematic reviews and all types of quantitative and qualitative study designs published in peer-reviewed scientific journals in the English language between November 1^st^ 2019 and October 4^th^ 2021. Conference abstracts, editorials, and personal communications were excluded. Studies were included if they reported (A) on the impact of COVID-19 on (B) at least one (≥1) health outcome (any type) on (C) the level of any type of ambulance care professional (e.g. nurse, paramedic, physician), and (D) if data for the ambulance care professionals was reported as subset within the article.

### Study selection

After deduplication of the search results, the articles were uploaded in Rayyan software [15]. The title and abstract selection round started with a team session to calibrate selection criteria by teamwise screening of the first twenty articles. Then, pairs of two reviewers (RE, TW, EWM, LV) independently screened the search results on title and abstract. After this selection round, remaining articles were screened full text, this round also started with a calibration session and ten articles were screened teamwise. Then, independent reviewers (RE, TW, EWM, LV) screened all included full texts. During both selection rounds, differences were discussed and resolved during weekly team sessions.

### Data extraction

The following study characteristics were extracted: publication year, setting, design, methods, type of professionals, type of health outcome, measurements. For studies that used valid instruments to measure health outcomes, significant predictors for these health outcomes were extracted also. In a study comparing ambulance care professionals with other healthcare professionals, significant different outcomes were extracted. Data were extracted by one independent researcher (RE, TW). After extraction, data were checked for completeness and correctness by a second researcher (RE, TW) If these data extractors disagreed, the difference was discussed and they tried to reach consensus.

### Quality assessment of included studies

To assess the quality of included studies, we used the JBI critical appraisal checklist for studies reporting prevalence data for the quantitative cross-sectional studies [16,17]. For the qualitative study we used the Critical Appraisal Skills Program (CASP) Checklist for qualitative research [16,18]. The quality assessment was performed by one researcher (RE) and validated by a second researcher (LV).

### Data synthesis and presentation

Due to the limited number of included studies and heterogeneity of populations, outcomes and instruments, a meta-analysis was not possible. Instead, we extensively analyzed and synthesized the studies by describing outcomes and impact in detail, as presented in the included studies.

## Results

### Review statistics

**Fig 1:**
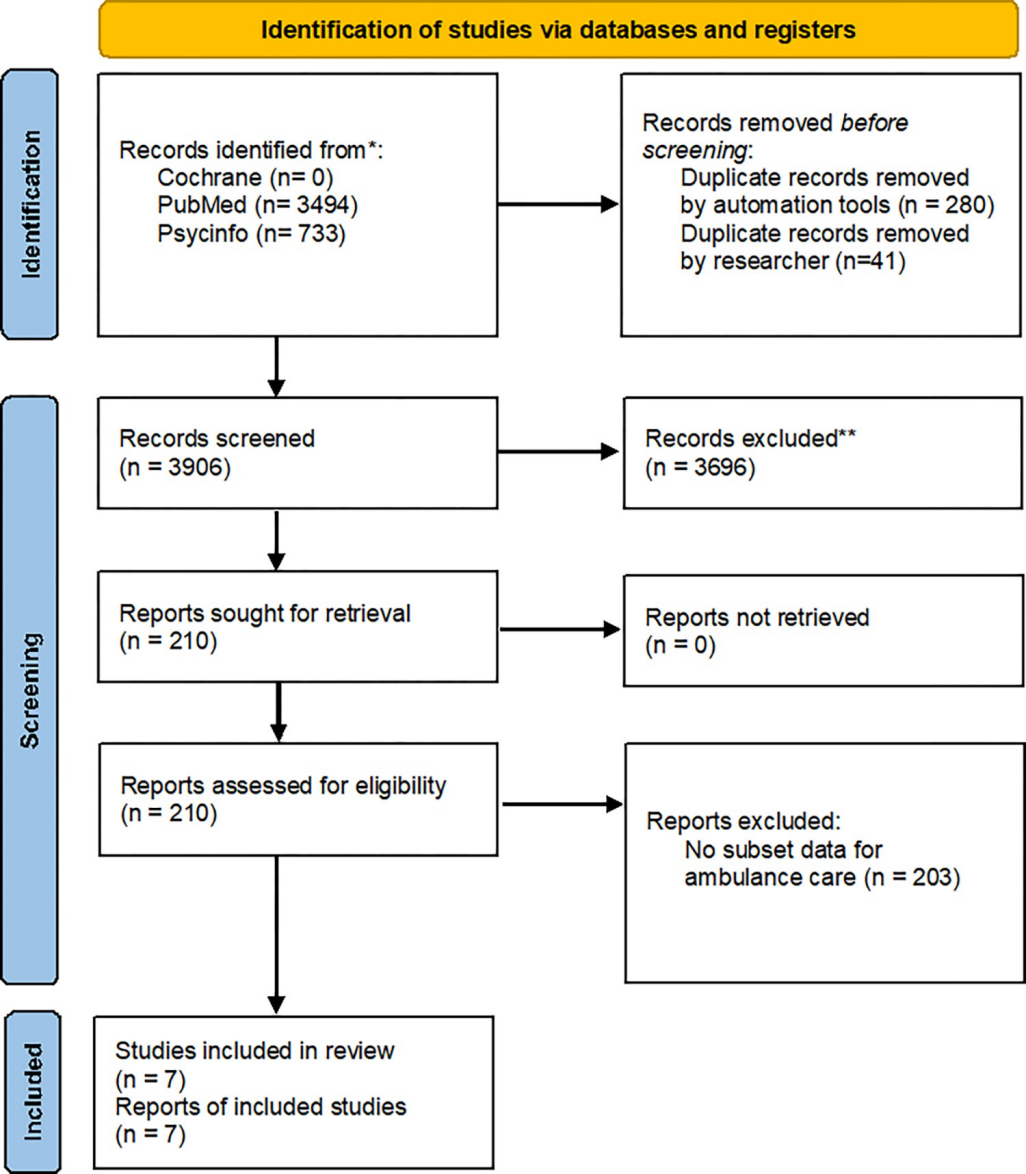


The initial searches resulted in 3906 unique hits and successive selection rounds identified seven articles meeting the selection criteria (Fig 1). During the full-text selection process 210 articles were screened and 203 were excluded as no subset for ambulance care was available.

### Study characteristics

The characteristics of the included studies (n = 7) are displayed in Table 1. The studies were conducted in Europe (n = 4) [19–22], and Asia (n = 3) [23–25]. Professionals included in these studies were ambulance care professionals (unspecified), paramedics, EMTs, and drivers. Six studies had a quantitative cross-sectional design and used surveys to collect data [19–22,24,25]. One study was qualitative and used semi-structured interviews [23].

**Table 1:** Characteristics included studies.

1^st^ author (year) Country (ref ID)	Aim	Design	Population (total and ambulance care)	Methods (data collection period)	Independent variables	Health outcomes
Abed Alah (2021) Qatar [25]	To determine the levels of PTSD, anxiety, and depression among healthcare workers and to explore the associated factors during the COVID-19 pandemic	Quantitative, cross-sectional	Total population (n = 394): physicians, nurses, technicians, paramedics Ambulance care professionals (n = 37): paramedics	Online survey (November-December 2020)	• 5 socio-demographic (age, gender, nationality, marital status, childrxsen) • 3 work-related (clinical experience, area of • work, profession)1 health (history of chronic diseases)3 infection (frequency of dealing with suspected or confirmed cases, status of PPE training, status of training for infectious outbreaks)	1. PTSD measured with revised Impact of Event Scale (IES-R) 2. Anxiety measured with Generalized Anxiety Disorder Questionnaire (GAD-7) 3. Depression measured with Patient Health Questionnaire (PHQ-9)
Dreher (2021) Germany [22]	To investigate attitudes, stressors and health outcomes and their potential determinants among ambulance care professionals and to detect possible changes within a 5-week period during the COVID-19 pandemic	Quantitative, cross-sectional	Ambulance care professionals (n = 1537): EMTs, paramedics	Online survey (April–May 2020)	• 5 socio-demographic (age, gender, marital status, children, highest level of education) • 4 infection (self-rated health, suspected or confirmed COVID-19 cases among friends and family, suspected or confirmed COVID-19 cases among colleagues, previous infection with COVID-19) • 4 occupational safety (feeling sufficiently prepared for COVID-19, perceived professional risk of infection, PPE availability, feeling sufficiently protected by PPE) • 2 work outcomes (workload, quality of care)	1. Anxiety measured with Generalized Anxiety Disorder Questionnaire (GAD-2) 2. Depression measured with Patient Health Questionnaire (PHQ-2) 3. Psychological burden related to COVID-19 (concern about infection during work, shortfall of colleagues, uncertainty of child care, uncertainty about how to act, contact persons, personal finances, letting patients down and the temporal scope of the crisis) measured with 8 self-formulated questions
Ilczak (2021) Poland [19]	To assess predictors of stress that paramedics, nurses and physicians experience during the COVID-19 pandemic	Quantitative, cross-sectional	Total population (n = 995): Paramedics, nurses and physicians working in emergency care Ambulance care professionals (n = 565): paramedics, nurses, physicians	Online survey developed and validated for the study (March-April 2020)	• 6 socio-demographic (age, gender, profession, place of work, work experience and education) • 18 questions on feelings and opinions	1. Stress measured with 1 self-formulated question on perceived stress
Martinez-Caballero (2021) Spain [20]	To analyze the impact of the COVID-19 pandemic on the mental health of ambulance care professionals	Quantitative, cross-sectional	Ambulance care professionals (n = 317): EMTs, nurses, physicians	Online survey (May-July 2020)	• 4 socio-demographic (age, gender, number of people living together during the pandemic, body weight) • 4 work-related (professional category, place of work, type of unit, experience, change of function) • 8 occupational safety (previous training in PPE usage, availability of PPE, non-compliance of equipment removal, information on the pandemic, testing for COVID-19, need for home isolation, presence of COVID-19 symptoms, hospitalization for COVID-19) • 8 variables associated with mental health (concerns about the possibility of contracting COVID-19, treatment of anxiety before and during the pandemic, need for psychological support before and during the pandemic, knowledge of the existence of a psychological support unit for employees, work environment of the unit, existence of specific training courses for anxiety control for workers)	1. Psychological distress measured with General Health Questionnaire (GHQ-12) 2. PTSD measured with Davidson Trauma Scale (DTS-8) 3. Insomnia measured with Athens Insomnia Scale (AIS-8) 4. Psychological burden related to COVID-19 (concern about infection during work, concern about transmission to home and other relatives) measured with 2 self-formulated questions 5. Anxiety measured with 1 self-formulated question
Munawar (2021) Pakistan [23]	To examine the psychological impact of COVID-19 on ambulance care professionals, their stress coping strategies or protective factors, and challenges while dealing with COVID-19 patients	Qualitative, framework thematic analysis	Ambulance care professionals (n = 15): EMTs, drivers	Semi-structured interviews (April 2020)	Topic list based on: 1. Expert opinion 2. Pre-interviews 3. Topic list and themes are not further described or available.	N/A
Nabe-Nielsen (2021) Denmark [21]	To compare COVID-19 risk management, fear of infection and fear of transmission of infection among frontline healthcare workers	Quantitative, cross-sectional	Frontline employees working within hospital/rehabilitation, eldercare, psychiatry, childcare and ambulance services (n = 2623) Ambulance care professionals (n = 140): (professions: unspecified)	Online survey (April 2020)	• 5 socio-demographic (age, gender, job title, area of work and region) • 1 insecurity regarding COVID-19 guidelines • 3 infection (exposure to infected patients, access to COVID-19 test, and access to PPE) • 4 communication and trust in workplace (clear communication, secure feeling about work organization during COVID-19 pandemic, workplace preparation, and attention to vulnerable employees)	1. Fear of infection and transmission of infection (fear of infection, fear of transmission to home and other relatives, and fear of transmission to clients/patients during work) measured with 3 self-formulated questions
Usul (2020 Turkey [24]	To determine the anxiety scores and related factors associated with the COVID-19 pandemic in ambulance care professionals	Quantitative, cross-sectional	Ambulance care professionals (n = 402): EMTs, paramedics, drivers, physicians, nurses	Online survey (period: unspecified)	• 5 socio-demographic (age, gender, marital status, number of children, family) • 2 work-related (profession, work experience)	1. Anxiety measured with State-Trait Anxiety Inventory state anxiety scale (STAI TX-1)

Abbreviations. EMT: Emergency Medical Technician, PPE: Personal protective equipment, PTSD: Post-traumatic stress disorder.

### Quality of included studies

The quality of the included studies is reported in Tables 2 and 3. The quality of the included quantitative designs was variable, with possible selection bias within sampling strategies and relatively small ambulance care sample sizes that represented small proportions of the total study populations. In addition, the validity of the methods was suboptimal, as not all studies used instrument that were systematically designed and tested (for validity) to measure health outcomes amongst ambulance care professionals. Instead, these studies used unvalidated self-formulated questions. The quality of the included qualitative study was low, as the recruitment strategy and data collection were not fully appropriate, and the relationship between the researcher and participants was not considered.

**Table 2:** Quality included qualitative study.

1^st^ author (Year) Country [ref ID]	Section A: Validity						Section B: Results			Section C: Valuableness
	1 Clear research aim	2 Appropriateness Qualitative methodology	3 Appropriateness design	4 Appropriateness recruitment strategy	5 Appropriateness data collection	6 Consideration relationship researcher–participants	7 Consideration ethical issues	8 Rigorousness data analysis	9 Clear statement findings	10 Valuableness results
Munawar (2021) Pakistan [23]	Y	Y	Y	N (unclear why these participants were the most appropriate to provide this knowledge)	N (topic list unclear)	N	Y	N (coding process unclear, contradictory data unclear)	N (credibility not discussed)	Y

Y = Yes, N = No,? = can’t tell.

**Table 3:** Quality included quantitative studies.

1^st^ author (Year) Country [ref ID]	1 Appropriateness sample frame	2 Appropriateness sample strategy	3 Adequate sample size	4 Detailed description of subjects and setting	5 Data analysis with sufficient coverage of sample	6 Validity methods for identification of the condition	7 Reliability condition measurement	8 Appropriateness statistical analysis	9 Adequate response management
Abed Alah (2021) Qatar [25]	Y	N (sampling though Red Cross, no direct access to EMS)	N (EMS sample was 9.4% of total)	Y	Y	Y	Y	Y	Y
Dreher (2021) Germany [22]	Y	N (sampling through professional organization)	N (EMS sample was 16% of total)	Y	Y	Y	Y	Y	Y
Ilczak (2021) Poland [19]	Y	N (sampling through professional organization)	N (EMS sample was 13.2% of total)	Y	Y	N (self-formulated question on outcome)	Y	Y	Y
Martinez-Caballero (2021) Spain [20]	Y	N (2 regions, possible selection bias)	Y	Y	Y	Y	Y	Y	Y
Nabe-Nielsen (2021) Denmark [21]	Y	N (sampling through professional organization)	N (EMS sample was 5,3% of total)	N (ambulance care professionals unspecified)	Y	N (self-formulated question on outcome)	Y	Y	Y
Usul (2020 Turkey [24]	U	U	U	Y	Y	Y	Y	Y	U

Y = Yes, N = No, U = Unclear, N/A = not applicable.

### Outcomes

Within the included studies, six different outcomes were quantitatively measured: stress/distress/PTSD, anxiety, depression, insomnia, fear, and psychological burden. Additionally, one study qualitatively explored coping with COVID-19 by ambulance care professionals.

### Stress/Distress/PTSD

Stress, distress and PTSD were measured in three studies [19,20,25]. Two studies used validated instruments: the General Health Questionnaire (GHQ-12), the Davidson Trauma Scale (DTS-8), and the 22-item revised Impact of Event Scale (IES-R) [20,25]. The prevalence of psychological distress was 36.0% and the prevalence of suspected PTSD (scores of clinical concern) varied from 18.5–30.9% [20,25]. The third study measured a 3.73 stress level on a 5-point Likert-scale with one self-formulated question on perceived stress [19]. There was a positive correlation between suspected PTSD and years of work experience (r = 0.133, p < 0.05) and a positive correlation between suspected PTSD and psychological distress (r = 0.622, p < 0.01) [20]. The prevalence of suspected PTSD was higher in paramedics compared to nurses (OR: 2.90, p = 0.037) [25].

### Anxiety

Anxiety was measured in four studies [20,22,24,25]. Two studies used a version of the validated Generalized Anxiety Disorder Questionnaire (GAD-2 and GAD-7) and reported a anxiety prevalence range of 14.2%-16.1% [22,25]. Another study used a validated State-Trait Anxiety Inventory (STAI) scale to measure state anxiety (STAI TX-1) and reported an average state anxiety score of 50.7 [24]. The fourth study measured anxiety with one self-formulated question in 65.6% of the professionals [20]. There was a negative correlation between state anxiety score and years of age (r = -0.139, p < 0.05) [24]. Furthermore, professionals who were concerned about infecting their family members had higher anxiety scores (52.1 versus 46.3) (p < 0.05); professionals who thought that they had adequate PPE had lower anxiety scores (48.4 versus 55.4) (p < 0.05); and women had higher anxiety scores (53.9) than men (47.8) (p < 0.05) [24]. The prevalence of anxiety symptoms was higher in paramedics compared to nurses (OR: 5.48, p = 0.002) [25].

### Depression

Depression was measured in two studies using a version of the validated Patient Health Questionnaire (PHQ-2 and PHQ-9) [22,25]. The prevalence range for depressive symptoms varied from 12.4%-15.3%.

### Insomnia

Insomnia was measured in one study using the validated the Athens Insomnia Scale (AIS-8), this study reported a 60.9% prevalence rate of suspected insomnia (scores of clinical concern) [20].

### Fear

One study measured fear of infection, fear of transmission to home and other relatives, and fear of transmission to patients during work, with 3 self-formulated questions [21]. The proportion of ambulance care professionals reporting fear of infection, fear of transmission to home and other relatives, and fear of transmission to patients was 49%, 68% and 41% respectively. Higher proportions of ambulance workers reported fear of infection (49%) and fear of transmission to home and other relatives (68%) compared to healthcare workers in eldercare, hospital, psychiatry and childcare (fear of infection: 40%, 38%, 30%, 27%; fear of transmission: 52%, 51%, 45%, 53% respectively) [21].

### Psychological burden

One study measured COVID-19 related psychological burden with eight self-formulated questions on burden related to possible infection during work, shortfall of colleagues, their child care situation, uncertainty about how to act, contact persons, their financial situation, letting patients down and the temporal scope of the crisis [22]. The proportion of ambulance care professionals experiencing these burdens was 49.4%, on average. Another study used two self-formulated questions on burden related to possible infection during work and concern about transmission to family members [20]. The proportion of ambulance care professionals experiencing these burdens was 92.2%, on average.

### Coping

One qualitative study provided in-depth insight how ambulance care professionals deal with COVID-19, which coping strategies they use, and which challenges they experience while taking care for patients with COVID-19 [23]. This study shows that ambulance care professionals use five different strategies to cope with COVID-19: limiting media exposure, limiting sharing of COVID-19 duty details, religious coping, conceptualizing COVID-19 as ‘just another emergency’, and having an empathic attitude towards COVID-19 patients.

## Discussion

This rapid review aimed to give an overview of the impact of COVID-19 on the reported mental health and well-being of ambulance care professionals and identified seven studies. This very low number of studies is remarkable in the light of findings from and high research output in other emergency care settings like the ED and ICU. For these settings, systematic reviews report on the high impact of COVID-19 on the health and well-being of (emergency) healthcare professionals, such as depression, anxiety, acute stress, PTSD, sleep disorders, and burnout [1–5,7–9]. Also, the ambulance care professional often is the first professional in contact with a (suspected) COVID-19 patient. Within this context, the ambulance care professional has to make decisions for treatment and conveyance on its own, is confronted with relatives in their own home environment, has to deal with limited time, and is exposed to the COVID-19 virus. Another factor that could contribute to the stress levels of ambulance care professionals related to work and COVID-19 could be the context of the households in which they reside related to the changes in patient’s socioeconomic conditions e.g. unemployment, and the circumstances because of COVID-19 and the lockdown. Yet, within literature there is little attention for this group of professionals during the COVID-19 pandemic.

Distress and PTSD prevalence rates, which were measured with validated instruments, are in range with other healthcare workers during COVID-19 [1,2,4,5], but higher compared to ambulance care professionals in the pre-COVID-19 era [10]. This increase can be related to ambulance care professionals being exposed to COVID-19 patients, as a previous meta-analysis shows that healthcare professionals in contact with patients infected with novel viruses show greater levels of PTSD and distress, compared to lower risk colleagues [26]. A remarkable finding was the prevalence rate of insomnia, although measured in one study. The insomnia prevalence measured in ambulance care professionals with a validated instrument was high compared to insomnia among healthcare workers during COVID-19 [3–5] and insomnia and sleeping problems among ambulance care professionals before COVID-19 [12,27,28]. Anxiety and depression prevalence rates that were measured with validated instruments are in range with other healthcare professionals during COVID-19 [1–5] and ambulance care professionals before COVID-19 [10]. Fear, psychological burden and stress could not be compared to literature.

Although our results provide a first insight in the health and well-being of ambulance care professionals during the COVID-19 pandemic, the included outcomes are too limited in number to draw strong conclusions, and do not adequately tap the concept of health or well-being of ambulance care professionals [11,29,30]. Studies examining similar outcomes as well as other types of health and well-being related outcomes (e.g. burnout, work engagement) are needed to further determine the actual impact of COVID-19 on the health and well-being of ambulance care professionals in a more holistic way.

Moreover, the included outcome variables in the identified studies are measured with a variety of instruments, ranging from internationally validated instruments to self-developed and unvalidated questionnaires. This limits comparability between populations and studies, and exposes the already under investigated ambulance care professional population to possibly less valid and reliable instruments. This urges the need to use validated and reliable instruments to measure a diversity of health outcomes to enable follow-up and comparability.

### Study implications and practical implication

Besides more epidemiological research to gain insight in the prevalence of health outcomes and predictors, there is also a need for qualitative research to gain in-depth understanding on the impact of COVID-19 on health and well-being of ambulance care professionals and coping strategies. In our study, only one qualitative study reporting on coping strategies was identified. Insight in coping strategies of ambulance care professionals is essential as dysfunctional coping styles might predict PTSD and functional coping styles may positively influence successful aging at work [31–33]. Also, in-depth understanding is necessary for the systematic development, testing and implementation of preventive and supportive tools and interventions, that match the variety of coping strategies used by ambulance care professionals [34].

While the impact of COVID-19 on the health and well-being of ambulance care professionals seems under investigated, the EMS field had focused on the impact of COVID-19 on early prehospital identification [35], EMS usage [36,37], effects on other patients groups like out-of-hospital cardiac arrest, heat stroke and substance abuse [38–40], transport [41], and on the knowledge of ambulance care professionals [42]. Our results urge a refocus of COVID-19 related prehospital research, and to include the health and well-being of ambulance care professionals as well.

### Strengths and limitations

Despite this rapid review being performed conform the international recommendations, this study has some limitations. The search was restricted to two databases, and was limited to the English language, therefore the search might have been less comprehensive compared to a full systematic review. Furthermore, parts of the selection and data extraction process were performed by one researcher, with a non-blinded quality check of a second reviewer. The included articles had biases on sample sizes, sample strategies, and valid measurements, and therefore have a variable quality.

## Conclusion

This rapid review shows that there is limited attention for the health and well-being of ambulance care professionals during the COVID-19 pandemic. Although the included number of studies and included outcomes are too limited to draw strong conclusions, our results indicate a significant impact of COVID-19 on the mental health of ambulance care professionals, with higher rates of distress, PTSD and insomnia compared to the pre-COVID-19 era. Besides the limited evidence, included studies also show variable quality, with health outcomes that are not always measured with validated and reliable instruments. Our results urge the need to investigate the health and well-being of ambulance care professionals during and after the COVID-19 pandemic.

## Supporting information

S1 Checklist PRISMA 2020 checklist. (DOCX)

S1 Appendix Appendix 1 –search strategies per database. (DOCX)

## Abbreviations

CASP: critical appraisal skills program
ED: emergency department
EMS: emergency medical service
EMT: emergency medical technician
ICU: intensive care units
OR: odds ratio
PPE: personal protective equipment
PTSD: post-traumatic stress disorder
