# Supplementary material for: The impact of COVID-19 on the mental health and well-being of ambulance care professionals: A rapid review
Source: PLoS One. 2023 Jul 11;18(7):e0287821. doi: 10.1371/journal.pone.0287821 (PMC10335670; doi:10.1371/journal.pone.0287821)
Supplement: S1 Appendix — (DOCX) [file pone.0287821.s002.docx]

**Appendix 1 – Search strategies per database**

**PubMed**

S1 "Emergency Medical Services"[Mesh] OR "Emergency Medicine"[Mesh] OR "Emergency Nursing"[Mesh] OR "Emergency Medical Technicians"[Mesh] OR emergency medical technician*[tiab] OR emergency medical personnel*[tiab] OR emergency medical staff[tiab] OR emergency medical service*[tiab] OR emergency personnel*[tiab] OR emergency staff[tiab] OR ambulance* [tiab] OR paramedic* [tiab] OR EMT [tiab] OR emergency nurs*[tiab] OR emergency medicine [tiab] OR emergency service*[tiab] OR emergency department[tiab] OR emergency room*[tiab] OR frontline[ti] OR Nurse*[ti] OR Healthcare provider*[ti] OR Healthcare worker*[ti] OR Healthcare staff*[ti] OR medical provider*[ti] OR medical worker*[ti] OR medical staff*[ti] OR first N1 responder*[ti]

#2 **"critical care nursing"[Mesh] OR “critical care”[Mesh] OR “intensive care units”[Mesh:NoExp] OR “Coronary Care Units”[MeSH] OR “Respiratory Care Units”[MeSH] OR acute care[tiab] OR critical care[tiab] OR intensive care[tiab] OR ICU[tiab] OR stroke unit*[tiab] OR coronary care[tiab] OR coronary unit*[tiab] OR respiratory care[tiab] OR respiratory unit*[tiab] OR ICU[tiab] OR IC[tiab]**

#3 #1 OR #2

#4 **("COVID-19"[tiab] OR "COVID-19"[MeSH] OR "SARS-CoV-2"[tiab] OR "sars-cov-2"[MeSH] OR "Severe Acute Respiratory Syndrome Coronavirus 2"[tiab] OR "NCOV"[tiab] OR "2019 NCOV"[tiab] OR (("coronavirus"[MeSH] OR "coronavirus"[tiab] OR "COV"[tiab] OR pandemic*[tiab] OR "middle east respiratory syndrome"[tiab] OR "severe acute respiratory syndrome"[tiab]) AND 2019/11/01[PDAT] : 3000/12/31[PDAT]))**

#5 #3 AND #4

#6 "Physical Endurance"[Mesh] OR "Physical Exertion"[Mesh] OR Fatigue[Mesh] OR "Sleepiness"[Mesh] OR "Sleep Initiation and Maintenance Disorders"[Mesh] OR "Sleep Deprivation"[Mesh] OR "physical endurance"[tiab] OR "Physical Exertion"[tiab] OR Exhaustion[tiab] OR fatigue[tiab] OR Sleepiness[tiab] OR "need for recovery"[tiab] OR powerlessness[tiab] OR "somatic complaints"[tiab] OR insomnia[tiab] OR Hypersomnia[tiab] OR "Sleep Deprivation"[tiab] OR "Physical health"[tiab] OR Physical impact[tiab]

#7 "Sick leave"[Mesh] OR "Workload"[Mesh] OR "Medical errors"[Mesh] OR "Work engagement"[Mesh] OR "Job Satisfaction"[Mesh] OR "Personnel Staffing and Scheduling"[Mesh] OR "sick leave"[tiab] OR Turnover[tiab] OR Absenteeism[tiab] OR "overtime work"[tiab] OR "Workload"[tiab] OR "work demand"[tiab] OR "work overload"[tiab] OR work pressure[tiab] OR "personal sacrifice"[tiab] OR "lack of concentration"[tiab] OR "Medical Errors"[tiab] OR blame[tiab] OR "Work Engagement"[tiab] OR "Job Satisfaction"[tiab] OR "Vocational health"[tiab] OR "Personnel Staffing and Scheduling"[tiab] OR workrelated[tiab]

#8 "Burnout, Professional"[Mesh] OR "Burnout, Psychological"[Mesh] OR burnout[tiab] OR burn out[tiab]

#9 "Trauma and Stressor Related Disorders"[Mesh] OR "Adjustment disorder" [tiab] OR posttraumatic stress[tiab] OR PTSD [tiab] OR PTSS [ tiab] OR "Psychological Trauma"[tiab] OR "moral injury"[tiab]

#10 "Adaptation, Psychological"[Mesh] OR "Psychosocial Functioning"[Mesh] OR "Coping"[tiab] OR "Emotional adjustment"[tiab] OR "Posttraumatic growth"[tiab] OR "Sense of coherence"[tiab] OR "Psychosocial Functioning"[tiab]

#11 "Attitude"[Mesh] OR "Catastrophization"[tiab] OR "Optimism"[tiab] OR "Pessimism"[tiab] OR "Respect"[tiab] OR "Stereotyping"[tiab]

#12 "Defense Mechanisms"[Mesh] OR "Life Style"[Mesh] OR "Acting Out"[tiab] OR "denial"[tiab] OR "Scapegoating"[tiab] OR "Projection"[tiab] OR "Rationalization"[tiab] OR Lifestyle[tiab]

#13 "Morale"[Mesh] OR "Ethics, Professional" [Mesh] OR "positive psychology" [Mesh] OR "spirituality" [Mesh] OR "Existentialism"[Mesh] OR "Morale"[tiab] OR "Moral distress"[tiab] OR "Moral dilemma"[tiab] OR "Ethical dilemma"[tiab] OR meaning*[tiab] OR "positive psychology"[tiab] OR spirituality[tiab] OR "Existentialism"[tiab] OR sense-making[tiab] OR striving[tiab] OR ideal*[tiab] OR "future prospects"[tiab] OR acceptance[tiab] OR re-attribution*[tiab] OR "causal understanding"[tiab] OR "identity"[tiab]

#14 "Work-Life Balance"[Mesh] OR "Social Support"[Mesh] OR "Social Isolation"[Mesh] OR "Psychosocial Deprivation"[Mesh] OR personal lives[tiab] OR "Work-Life Balance"[tiab] OR "Social Support"[tiab] OR "Social Isolation"[tiab] OR "Psychosocial Deprivation"[tiab]

#15 #6 OR #7 OR #8 OR #9 OR #10 OR #11 OR #12 OR #13 OR #14

#16 #5 AND #15

#17 Emotions[mesh] OR "affect"[tiab] OR "Anger"[tiab] OR "Anxiety"[tiab] OR "apathy" [tiab] OR "Bereavement"[tiab] OR "Boredom"[tiab] OR "Disgust"[tiab] OR "Fear"[tiab] OR "Frustration"[tiab] OR "Guilt"[tiab] OR Shame[tiab] OR "Hate"[tiab] OR "Hostility"[tiab] OR "jealousy"[tiab] OR "Loneliness"[tiab] OR "Sadness"[tiab] OR "Critical incident stress"[tiab] OR "Emotional strain"[tiab] OR "Emotional burden"[tiab] OR "emotional labor"[tiab] OR "nervousness"[tiab] OR insecurity[tiab] OR "loss of confidence"[tiab]

#18 "Denial, Psychological"[Mesh] OR "Self Efficacy"[Mesh] OR "Self-Neglect"[Mesh] OR "Psychological Distress"[Mesh] OR "Mental Disorders"[Mesh] OR "Depression"[Mesh] OR "Stereotyping"[Mesh] OR "Social Stigma"[Mesh] OR "Suicide"[Mesh] OR "Occupational Stress"[Mesh] OR "Mental Health"[Mesh] OR "Stress, Psychological"[Mesh] OR "Rumination, Cognitive"[Mesh] OR "Resilience, Psychological"[Mesh] OR "Mental Health"[Mesh] OR "Denial"[tiab] OR "Self Efficacy"[tiab] OR "Self-Neglect"[tiab] OR "Burnout"[tiab] OR threat[tiab] OR "Psychological Distress"[tiab] OR "Mental Disorders"[tiab] OR "Depression"[tiab] OR "Stereotyping"[tiab] OR "Suicide"[tiab] OR "psychological adaption"[tiab] OR "Occupational Stress"[tiab] OR "Mental Health"[tiab] OR "Psychological stress"[tiab] OR "Rumination"[tiab] OR "Resilience"[tiab] OR "Mental Health"[tiab]

#19 "Pain"[Majr] OR "Headache"[Mesh]OR pain*[tiab] OR ache*[tiab] OR Headache*[tiab] OR Cephalgia*[tiab] OR Cephalalgia*[tiab]

#20 #17 OR #18 OR #19

#21 Worker*[ti] OR nurs*[ti] OR staff*[ti] OR professional*[ti] OR doctor*[ti] OR physician*[ti] OR employee*[ti]

#22 #20 AND #21

#23 #5 AND #22

#24 #16 OR #23

**PsycInfo**

S1 DE "Emergency Personnel" OR DE "First Responders" OR DE "Paramedics" OR DE "Emergency Medicine" OR DE "Emergency Services" OR DE "Crisis Intervention Services" OR TI (frontline OR Nurse* OR ((Healthcare OR medical) N1 (provider* OR worker* OR staff*)) OR (first N1 responder*) OR (emergency N3 (service* OR technician* OR personnel OR staff* OR room* OR nurs* OR department*)) OR ambulance* OR paramedic* OR EMT OR (emergency W0 (nursing OR medicine OR service*))) OR AB ((emergency N3 (service* OR technician* OR personnel OR staff* OR room* OR nurs* OR department*)) OR ambulance* OR paramedic* OR EMT OR (emergency W0 (nursing OR medicine OR service*))) OR SU (frontline OR Nurse* OR ((Healthcare OR medical) N1 (provider* OR worker* OR staff*)) OR (first N1 responder*) OR (emergency N3 (service* OR technician* OR personnel OR staff* OR room* OR nurs* OR department*)) OR ambulance* OR paramedic* OR EMT OR (emergency W0 (nursing OR medicine OR service*)))

S2 DE "Intensive Care" OR DE "Neonatal Intensive Care" OR TI (((acute OR critical OR intensive OR coronary OR respiratory) N1 (care OR unit*)) OR ICU OR guci OR IC OR nicu) OR AB (((acute OR critical OR intensive OR coronary OR respiratory) N1 (care OR unit*)) OR ICU OR guci OR IC OR nicu) OR OR SU (((acute OR critical OR intensive OR coronary OR respiratory) N1 (care OR unit*)) OR ICU OR guci OR IC OR nicu)

S3 S1 OR S2

S4 DE "COVID-19" OR **TI ("COVID-19" OR "SARS-CoV-2" OR "Severe Acute Respiratory Syndrome Coronavirus 2" OR NCOV) OR AB ("COVID-19" OR "SARS-CoV-2" OR "Severe Acute Respiratory Syndrome Coronavirus 2" OR NCOV) OR SU ("COVID-19" OR "SARS-CoV-2" OR "Severe Acute Respiratory Syndrome Coronavirus 2" OR NCOV)**

**S5 (DE "Coronavirus" OR TI ("coronavirus" OR "corona virus" OR COV OR pandemic* OR "middle east respiratory syndrome" OR "severe acute respiratory syndrome") OR AB ("coronavirus" OR "corona virus" OR COV) OR SU ("coronavirus" OR "corona virus" OR COV OR pandemic* OR "middle east respiratory syndrome" OR "severe acute respiratory syndrome")) AND Limiters - Published Date: 20191101-20211231**

**S6 S4 OR S5**

**S7 S3 AND S6**
